# Ovarian Cancer Cell-Conditioning Medium Induces Cancer-Associated Fibroblast Phenoconversion through Glucose-Dependent Inhibition of Autophagy

**DOI:** 10.3390/ijms25115691

**Published:** 2024-05-23

**Authors:** Alessandra Ferraresi, Carlo Girone, Chinmay Maheshwari, Letizia Vallino, Danny N. Dhanasekaran, Ciro Isidoro

**Affiliations:** 1 Laboratory of Molecular Pathology, Department of Health Sciences, Università del Piemonte Orientale, Via Solaroli 17, 28100 Novara, Italy; carlo.girone@uniupo.it (C.G.); chinmay.maheshwari@uniupo.it (C.M.); letizia.vallino@uniupo.it (L.V.); 2 Stephenson Cancer Center, The University of Oklahoma Health Sciences Center, Oklahoma City, OK 73104, USA; danny-dhanasekaran@ouhsc.edu

**Keywords:** autophagy, tumor microenvironment, ovarian cancer, reverse Warburg effect, metabolic reprogramming, hexokinase 2, resveratrol, cancer cell secretome

## Abstract

One aspect of ovarian tumorigenesis which is still poorly understood is the tumor–stroma interaction, which plays a major role in chemoresistance and tumor progression. Cancer-associated fibroblasts (CAFs), the most abundant stromal cell type in the tumor microenvironment, influence tumor growth, metabolism, metastasis, and response to therapy, making them attractive targets for anti-cancer treatment. Unraveling the mechanisms involved in CAFs activation and maintenance is therefore crucial for the improvement of therapy efficacy. Here, we report that CAFs phenoconversion relies on the glucose-dependent inhibition of autophagy. We show that ovarian cancer cell-conditioning medium induces a metabolic reprogramming towards the CAF-phenotype that requires the autophagy-dependent glycolytic shift. In fact, 2-deoxy-D-glucose (2DG) strongly hampers such phenoconversion and, most importantly, induces the phenoreversion of CAFs into quiescent fibroblasts. Moreover, pharmacological inhibition (by proline) or autophagy gene knockdown (by siBECN1 or siATG7) promotes, while autophagy induction (by either 2DG or rapamycin) counteracts, the metabolic rewiring induced by the ovarian cancer cell secretome. Notably, the nutraceutical resveratrol (RV), known to inhibit glucose metabolism and to induce autophagy, promotes the phenoreversion of CAFs into normal fibroblasts even in the presence of ovarian cancer cell-conditioning medium. Overall, our data support the view of testing autophagy inducers for targeting the tumor-promoting stroma as an adjuvant strategy to improve therapy success rates, especially for tumors with a highly desmoplastic stroma, like ovarian cancer.

## 1. Introduction

Ovarian cancer incidence has been increasing in recent decades, and this trend is expected to persist due to the increased average life expectancy and exposure to risk factors. This poses an urgency in research for improving early diagnosis, treatment efficacy, and patients’ quality of life [1]. The desmoplastic stroma, accounting for up to or more than 50% of the ovarian tumor mass, contributes to therapy failure since the stromal physical barrier hinders the delivery of anti-cancer drugs, and this correlates with poor prognosis [2]. The stromal composition (in terms of vascular structures, cellular components, and secreted factors) and its dynamic changes play a fundamental role in cancer initiation and progression [3]. Depending on its composition, the stroma can either prevent the neoplastic transformation or sustain the proliferation and spread of cancer cells [4]. For instance, benign epithelial tumors are surrounded by a tumor-restraining stroma that obstructs the spread of cancer cells in the adjacent environment, increasing cell adhesion to the extracellular matrix (ECM) [5] and thus limiting/preventing the malignant transformation of the epithelial cells [6]. On the other hand, tumor-generated paracrine signaling reshapes the microenvironment toward a tumor-promoting stroma characterized by immunosuppressive and angiogenic features that promote the emergence of chemoresistance and metastasis [7,8].

Cancer-associated fibroblasts (CAFs) represent the main type of stromal cells and consist of a heterogeneous and plastic population that differ in their origin, phenotype, function, and presence in different tumors [9,10]. CAFs can originate from a variety of cells and through different processes such as the phenoconversion of resident fibroblasts (which appears to be the primary route), the differentiation of precursor cells (e.g., mesenchymal stem cells), the de-differentiation of mature cells (e.g., adipocytes, pericytes, and stellate cells), or the trans-differentiation of cells (e.g., endothelial cells and epithelial cancer cells) in response to different direct, short-, and long-range paracrine signals [11,12]. The metabolic reprogramming towards aerobic glycolysis is a key functional feature that distinguishes CAFs from normal fibroblasts [13,14]. Glycolytic reprogramming in CAFs leads to the synthesis and secretion of energy-rich ‘fuels’ (such as lactate, pyruvate, ketone bodies, and fatty acids) that, in turn, feed cancer cells and sustain their increased anabolic needs (a process that has been named “reverse Warburg effect”) [15,16]. 

Autophagy, an evolutionary conserved catabolic mechanism, has been proven to shape the stroma composition (reviewed in [17]) and, by doing so, to affect ovarian cancer progression (reviewed in [18]). Recently, several studies aimed at identifying an autophagy-related signature able to predict cancer progression and therapy response. So far, Chen et al. identified seven autophagy-related genes (ULK2, ATG5, GABARAPL1, ATG4C, ATG12, ATG4A, and IFNG) for the stratification of ovarian cancer patients in high-risk and low-risk groups and associated with the modulation of tumor microenvironment [19]. Similarly, Ding et al. reported a molecular signature including eight autophagy-hub genes (ZFYVE1, AMBRA1, LAMP2, TRAF6, PDPK1, ATG2B, DAPK1, and TP53INP2) that correlates with the immune score (memory B cells, CD4 and CD8 T cells, and macrophages) of ovarian cancer patients [20]. Autophagy also plays a critical role in CAFs formation and tumor–stroma crosstalk by participating in the metabolic reprograming of CAFs [17,21].

Here, we demonstrate that ovarian cancer cell secretions promote the conversion of normal fibroblasts (NFs) into CAFs by inducing such a glycolytic shift that is dependent on the modulation of autophagy. In fact, this phenotypic and metabolic reprogramming is inhibited by the glycolytic inhibitor 2-deoxy-D-glucose (2DG), and exacerbated by the inhibition of the autophagy pathway; conversely, activators of autophagy hamper the phenoconversion and even attenuate the CAF-phenotype to a milder, normal-like phenotype. In a similar fashion, we show that the nutraceutical resveratrol (RV), that reduces the glucose uptake and concomitantly upregulates autophagy, can promote the CAFs phenoreversion. 

The present findings have clinical implications, supporting the use of autophagy inducers (e.g., nutraceuticals or small molecules) for targeting the tumor-promoting stroma to increase anti-cancer therapy efficacy and thus improve patients’ clinical outcomes.

## 2. Results

### 2.1. The Secretion from Different Ovarian Cancer Cells Promotes the Phenoconversion of Normal Skin Fibroblasts (NFs) into Cancer-Associated Fibroblasts (CAFs)

**Figure 1:**
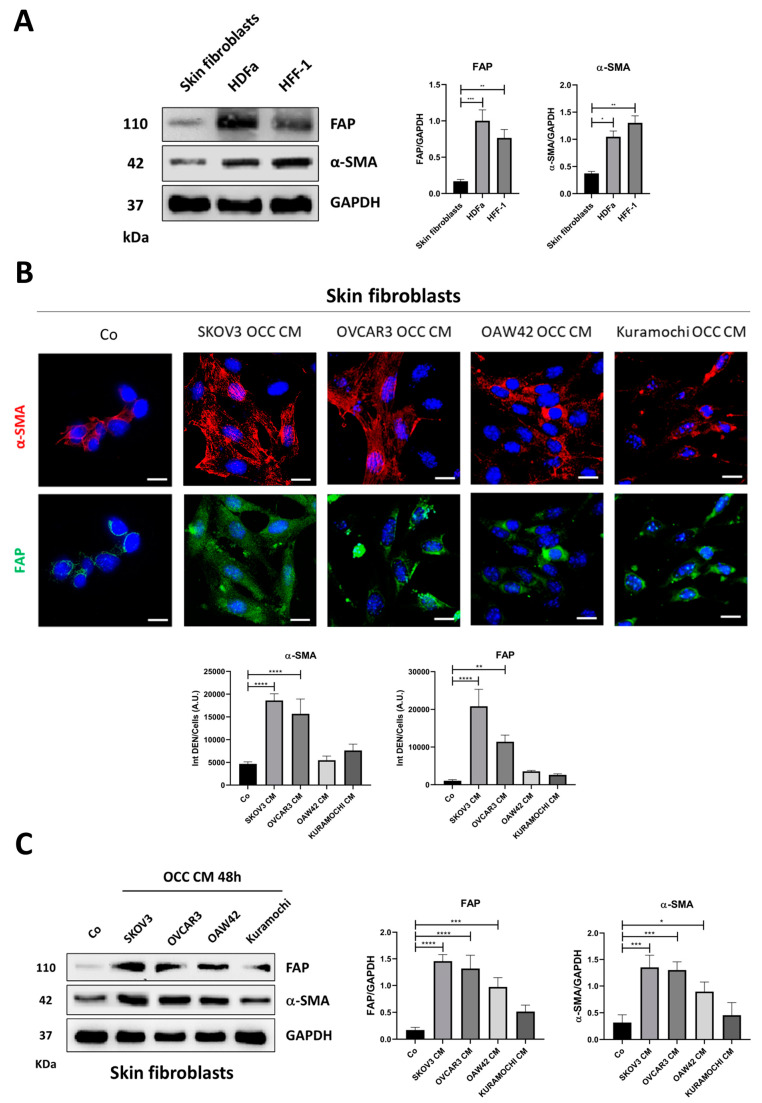
Ovarian cancer cell-conditioning medium (OCC CM) induces the phenoconversion of normal skin fibroblasts to CAFs. (**A**) Skin fibroblasts and HDFa and HFF-1 cell homogenates were analyzed by means of Western blotting for the expression of the CAF-markers FAP and α-SMA. The filter was probed with GAPDH as a loading control. (**B**,**C**) Skin fibroblasts were cultured with control medium (Co) or OCC CM collected from four different cancer cell lines (SKOV3, OVCAR3, OAW42, and Kuramochi) for 48 h. (**B**) Cells were fixed and stained for α-SMA (red) and FAP (green). The images were acquired with a fluorescence microscope and are representative of different fields per each condition. The average fluorescent integrated signal was determined using the ImageJ tool as described in the Section 4. Scale bar = 20 μm; magnification = 63×. (**C**) Cell homogenates were analyzed by means of Western blotting for the expression of FAP and α-SMA. The filter was probed with GAPDH as a loading control. The Western blotting is representative of three replicates, and densitometric data are reported in the graphs. Statistical analysis was performed using GraphPad Prism 5.0 software. Bonferroni’s multiple comparison test after one-way ANOVA analysis (unpaired, two-tailed) was employed. Significance was considered as follows: **** *p* < 0.0001; *** *p* < 0.001; ** *p* < 0.01; * *p* < 0.05.

First, we tested the ability of the conditioning medium from ovarian cancer cell lines (SKOV3, OVCAR3, OAW42, and Kuramochi), with a different profile of relevant oncogenes and oncosuppressor genes (see Section 4), to induce the CAF-phenotype in three different fibroblast cell lines (skin fibroblasts, HDFa, and HFF-1). We checked the basal expression of two important CAF-specific markers, FAP and α-SMA, both frequently upregulated in ovarian CAFs isolated from patients’ tumor specimens [22]. As shown in Figure 1A, the expression of FAP and α-SMA is much higher in HDFa and HFF-1 cells than in skin fibroblasts (Figure 1A). 

The modulation of CAF-markers in skin fibroblasts was then checked after 48 h incubation with ovarian cancer cell-conditioning medium (OCC CM).

OCC CM from SKOV3, and with lesser efficiency from OVCAR3 and OAW42 cells, was able to induce the CAF-phenotype in skin fibroblasts, with the formation of a stress fiber pattern typical of myofibroblasts and the significant upregulation of FAP and α-SMA, as shown by means of immunofluorescence and Western blotting (Figure 1B,C). OCC CM from Kuramochi cells elicited a slight increase in the expression of CAF-markers, although not statistically significant (Figure 1B,C). 

**Figure 2:**
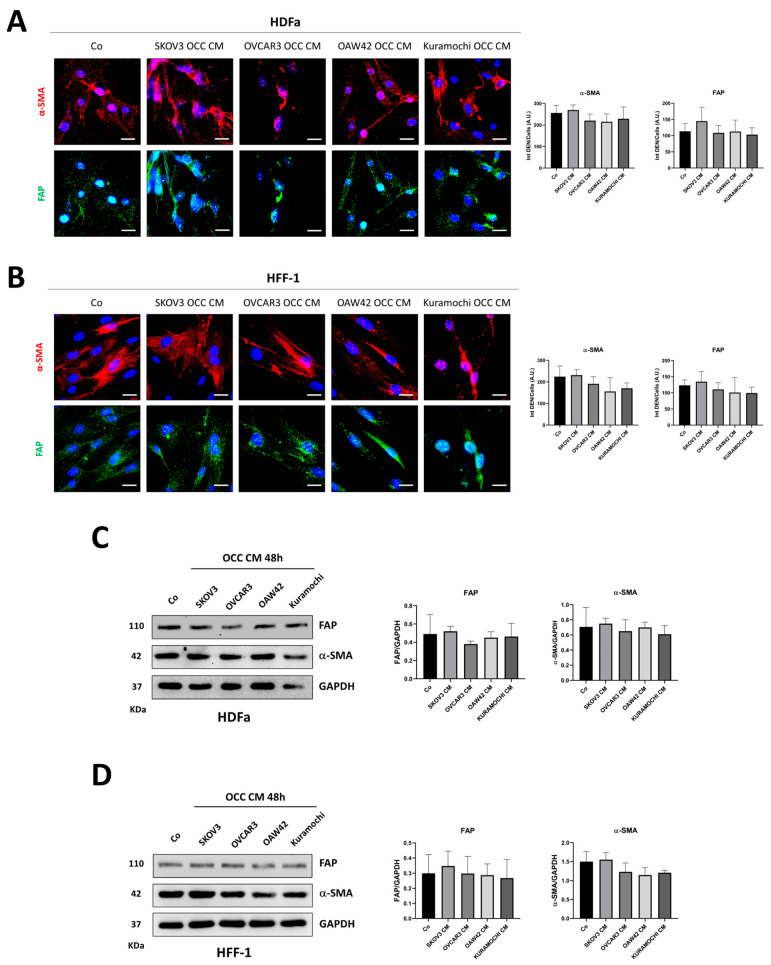
HDFa and HFF-1 do not undergo phenoconversion to CAFs when cultured with ovarian cancer cell secretions. HDFa and HFF-1 cells were cultured with control medium (Co) or OCC CM collected from four different cancer cell lines (SKOV3, OVCAR3, OAW42, and Kuramochi) for 48 h. (**A**,**B**) HDFa and HFF-1 cells were fixed and stained for α-SMA (red) and FAP (green). The images were acquired with a fluorescence microscope, and the quantification of fluorescence signals is reported in the histograms. Scale bar = 20 μm; magnification = 63×. (**C**,**D**) HDFa and HFF-1 cell homogenates were analyzed by means of Western blotting for the expression of FAP and α-SMA. The filters were probed with GAPDH as a loading control. The densitometric data of the triplicates are reported in the graphs. Statistical analysis was performed using GraphPad Prism 5.0 software. Bonferroni’s multiple comparison test after one-way ANOVA analysis (unpaired, two-tailed) was employed. The statistical analysis performed does not reveal any significance in the comparisons assessed.

Next, we questioned whether the cancer cell-conditioning medium may elicit similar effects on the phenoconversion of the other two fibroblast cell lines. Surprisingly, this effect was not reproduced in HDFa nor in HFF-1 cells (Figure 2), likely due to their higher basal expression of FAP and α-SMA compared to that observed in skin fibroblasts (Figure 1A). 

To study the molecular mechanisms involved in CAFs activation, in subsequent experiments, we used the secretome from the SKOV3 cell line and the skin fibroblasts as a cell model (henceforth referred to as normal fibroblasts, NFs).

### 2.2. CAFs Phenoconversion Requires the Glycolytic Shift and Can Be Reversed through Glycolysis Inhibition

**Figure 3:**
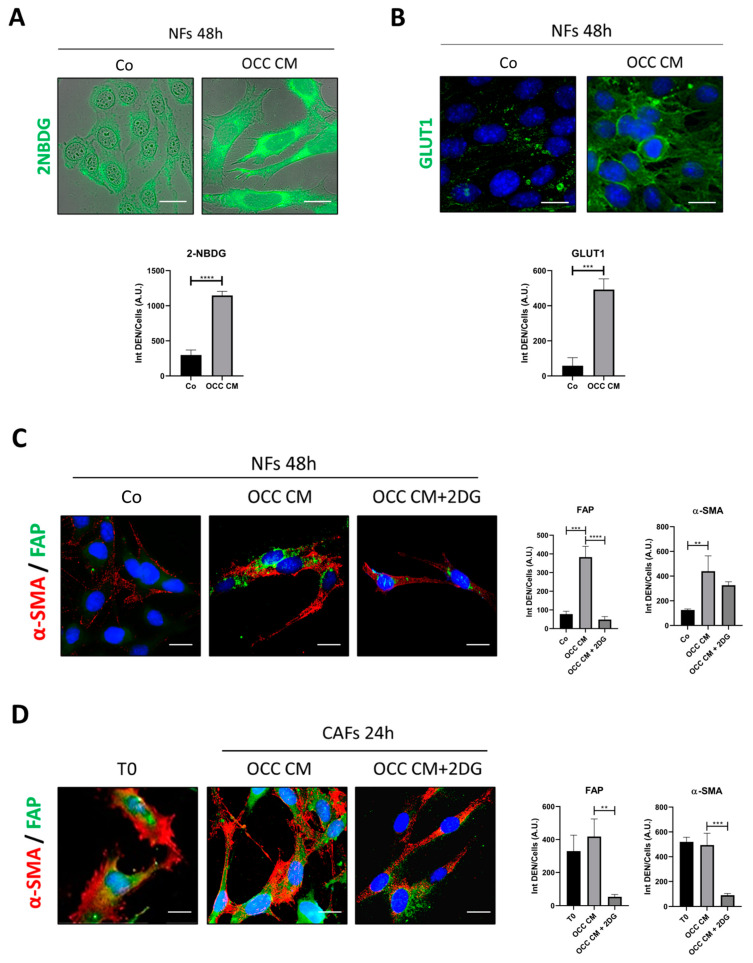
Inhibition of glycolysis not only counteracts the phenoconversion of NFs to CAFs but also converts CAFs back to NFs. (**A**,**B**) NFs cultured with Co or OCC CM for 48 h. (**A**) Glucose uptake monitored by means of 2-NBDG green dye internalization. The quantification of 2-NBDG fluorescence intensity/cells is reported in the graph. (**B**) Immunofluorescence for monitoring GLUT1 (green) subcellular localization. The fluorescence quantification of GLUT1 expression is reported in the graph. (**C**) NFs treated with Co, OCC CM, or OCC CM + 2DG for 48 h. Immunofluorescence double-staining for α-SMA (red)—FAP (green). The fluorescence quantification of the expression of FAP and α-SMA is reported in the graphs. (**D**) Previously obtained CAFs (T0) were cultured with OCC CM or OCC CM + 2DG for 24 h. Immunofluorescence double-staining for α-SMA (red)—FAP (green). The fluorescence quantification of the expression of FAP and α-SMA is reported in the graphs. The images shown are representative of different fields for each condition from three independent experiments. Average fluorescence intensity was calculated from at least 50 cells from randomly chosen fields. Scale bar = 20 μm; magnification = 63×. Statistical analysis was performed using GraphPad Prism 5.0 software. T-test analysis (unpaired, two-tailed) was employed in the panels (**A**,**B**) that required the comparison of two groups (control vs. treatment), while Bonferroni’s multiple comparison test after one-way ANOVA analysis (unpaired, two-tailed) was employed for panels (**C**,**D**). Significance was considered as follows: **** *p* < 0.0001; *** *p* < 0.001; ** *p* < 0.01.

The pivotal role of glycolysis in the metabolic reprogramming of CAFs is well established [13,23,24]. We assayed the glucose consumption in fibroblasts by monitoring the uptake of 2-NBDG, a fluorescent analog of glucose, along with the subcellular localization of GLUT1. Under the condition that produced the CAFs conversion (Figure 1B,C), the internalization of 2-NBDG and the plasma membrane translocation of GLUT1 were increased (Figure 3A,B). To prove that glucose uptake and glycolysis are instrumental to CAFs activation, we employed 2-deoxyglucose (2DG), a metabolically inert glucose analog acting as a competitive inhibitor of hexokinase 2 (HK2), the first rate-limiting step of glycolysis, that impairs glucose metabolism [25]. The addition of 2DG to OCC CM markedly prevented the phenoconversion into CAFs, as indicated by the decreased expression of α-SMA and FAP and the partial loss of the contractile morphology, a pattern resembling that of normal fibroblasts (NFs) incubated with control medium (Co) (Figure 3C). More importantly, when CAFs (produced as in Figure 3C) were switched into a culture with OCC CM supplemented with 2DG, their phenotype (partially) reverted to normal-like fibroblasts in just 24 h (Figure 3D). It should be noted that the CAFs cultured in control medium (Co) lose their activated phenotype within 48–96 h (Supplementary Figure S1), suggesting that the continuous presence of cancer-derived mediators and metabolites is necessary for maintaining the CAFs’ epigenetic changes. From these data, we conclude that the glycolytic shift is an early event mandatory for priming the NF-to-CAF-transition and necessary for maintaining the CAF-phenotype, and that its inhibition can revert the CAF-phenotype.

### 2.3. Autophagy Opposes the Transition towards CAFs

**Figure 4:**
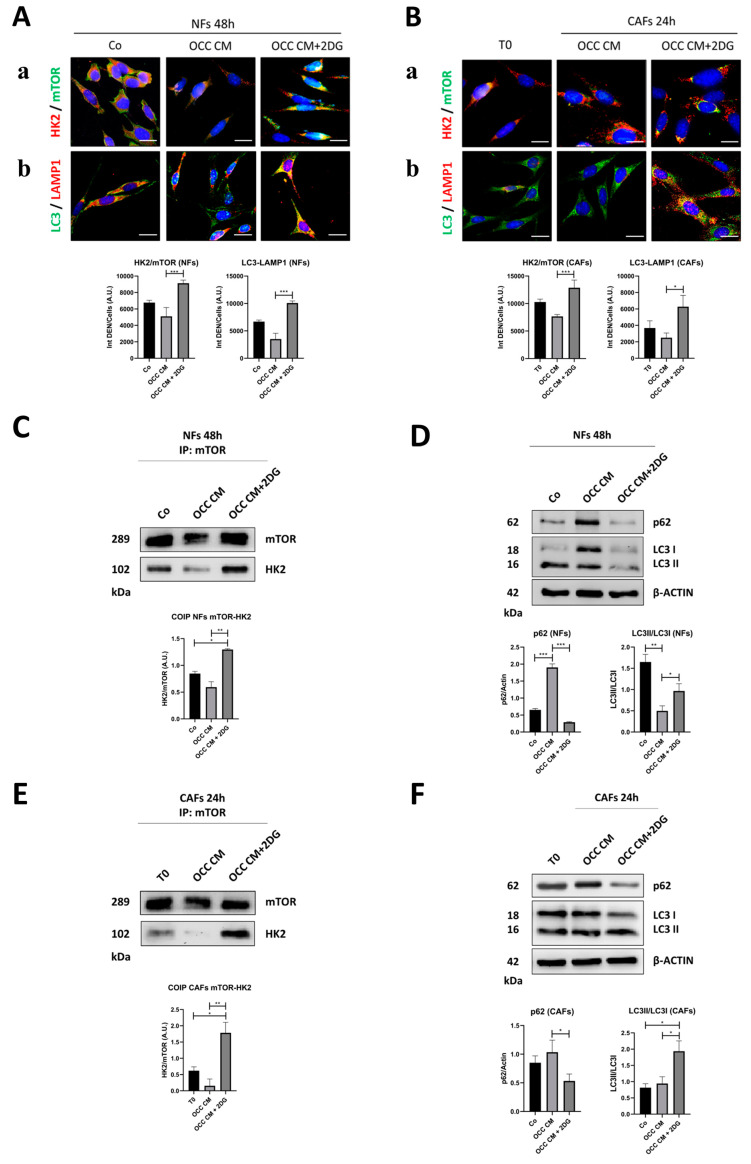
Glycolysis inhibition by 2DG promotes mTOR-HK2 interaction that, in turn, results in autophagy induction. (**A**) NFs were cultured with Co, OCC CM, or OCC CM + 2DG for 48 h. Immunofluorescence double staining for glycolysis/autophagy switch HK2 (red)—mTOR (green) (**a**) and LC3 (green)—LAMP1 (red) (**b**). The fluorescence quantification of (**a**) HK2-mTOR and (**b**) LC3-LAMP1 co-localization is reported in the graphs. Scale bar = 20 μm; magnification = 63×. (**B**) Previously obtained CAFs (T0) were cultured with OCC CM or OCC CM + 2DG for 24 h. Immunofluorescence double staining for glycolysis/autophagy switch HK2 (red)—mTOR (green) (**a**) and LC3 (green)—LAMP1 (red) (**b**). The fluorescence quantification of (**a**) HK2-mTOR and (**b**) LC3-LAMP1 co-localization is reported in the graphs. Scale bar = 20 μm; magnification = 63×. (**C**,**D**) NFs were cultured as described in panel A. Cell homogenates were processed using immunoprecipitation to assess mTOR-HK2 interaction (**C**) and Western blotting for the expression of the autophagy markers p62 and LC3 (**D**). (**E**,**F**) Previously obtained CAFs (T0) were cultured with OCC CM or OCC CM + 2DG for 24 h. Cell homogenates were analyzed as detailed for NFs to assess mTOR-HK2 interaction (**E**) and the expression of p62 and LC3 (**F**). The densitometric data are reported in the graphs. Statistical analysis was performed using GraphPad Prism 5.0 software. Bonferroni’s multiple comparison test after one-way ANOVA analysis (unpaired, two-tailed) was employed. Significance was considered as follows: *** *p* < 0.001; ** *p* < 0.01; * *p* < 0.05.

We have previously shown that IL-6-induced glucose uptake results in autophagy inhibition in OCCs [26]. Here, we investigated whether a link between glucose consumption and autophagy could underly the metabolic reprogramming regulating the NF-to-CAF transition. Hexokinase-2 (HK2), an enzyme that catalyzes the first step of glycolysis, functions as a molecular switch to trigger autophagy by inhibiting mTORC1 (mammalian target of rapamycin complex 1), the key negative regulator of autophagy [26,27]. We analyzed the co-localization of HK2 and mTOR using immunofluorescence co-labeling. When NFs were incubated for 48 h in OCC CM, which results in increased glucose uptake and phenoconversion into CAFs (Figure 3A–C), mTOR fluorescent signal was barely detectable and no co-staining with HK2 was observed, whereas the addition of 2DG, which prevents the phenoconversion into CAFs, enforced the close proximity of the two proteins (Figure 4A panel a). When the CAFs were incubated for 24 h with 2DG, which attenuates/reverses their phenotype, HK2 and mTOR co-localized (Figure 4B panel a). Since, in this case, mTOR cannot inhibit autophagy, we monitored autophagy induction by means of LC3 (marker of autophagosomes)—LAMP1 (marker of lysosomes) co-staining. The addition of 2DG to OCC CM resulted in a marked increase in yellow signal, indicative of autolysosome formation, both in NFs and CAFs (Figure 4A,B, panel b). The interaction between mTOR and HK2 was confirmed through immunoprecipitation. As shown in Figure 4C–E, the addition of 2DG to OCC CM significantly promoted the interaction of the two proteins. Autophagy induction by 2DG was further confirmed by means of Western blotting (Figure 4D–F), as indicated by decreased levels of p62 (indicative of enhanced cargo degradation) and an increased LC3-II/LC3-I ratio (indicative of the maturation of the autophagosomal marker LC3). 

**Figure 5:**
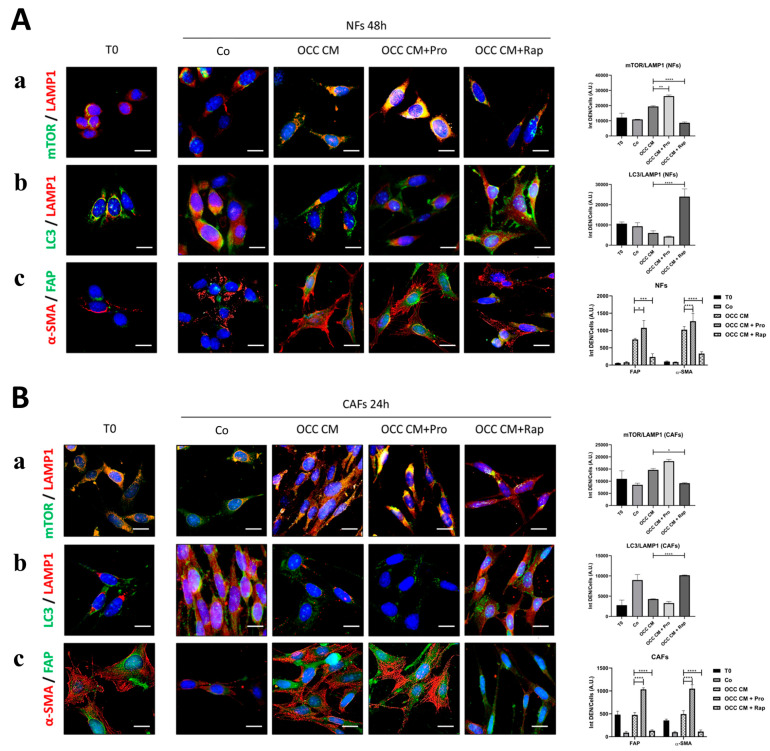
Autophagy counteracts NF-to-CAF transition and reprograms CAFs towards NFs. (**A**) NFs were cultured with Co, OCC CM, OCC CM + Pro, or OCC CM + Rap for 48 h. Immunofluorescence double-staining for (**a**) mTOR-lysosomal detachment (mTOR (green)—LAMP1 (red)), (**b**) autolysosome formation (LC3 (green)—LAMP1 (red)), and (**c**) phenoconversion (FAP (green)—α-SMA (red)). The fluorescence quantification of (**a**) mTOR-LAMP1, (**b**) LC3-LAMP1 co-localization, and the expression of (**c**) FAP and α-SMA is reported in the graphs. (**B**) Previously obtained CAFs (T0) were cultured with Co, OCC CM, OCC CM + Pro, or OCC CM + Rap for 24 h. Immunofluorescence double-staining for (**a**) mTOR-lysosomal localization (mTOR (green)—LAMP1 (red)), (**b**) autolysosome formation (LC3 (green)—LAMP1 (red)), and (**c**) phenoconversion (FAP (green)—α-SMA (red)). The quantification of (**a**) mTOR-LAMP1, (**b**) LC3-LAMP1 co-localization, and the expression of (**c**) FAP and α-SMA is reported in the graphs. The images shown are representative of different fields for each condition from three independent experiments. Average fluorescence intensity was calculated from at least 50 cells from randomly chosen fields. Scale bar = 20 μm; magnification = 63×. Statistical analysis was performed using GraphPad Prism 5.0 software. Bonferroni’s multiple comparison test after one-way ANOVA analysis (unpaired, two-tailed) was employed. Significance was considered as follows: **** *p* < 0.0001; *** *p* < 0.001; ** *p* < 0.01; * *p* < 0.05.

We hypothesized that autophagy induction can promote the reversion of the CAF-phenotype. To corroborate this hypothesis, OCC CM was supplemented with proline (Pro) or rapamycin (Rap) to induce or inhibit mTOR activity, respectively [28,29]. In NFs incubated with OCC CM, mTOR co-localized with LAMP1, indicative of active mTORC1 and the inhibition of autophagy, as shown by the low level of LC3 and of its co-localization with LAMP1 (Figure 5A, panels a,b). When proline was added to the medium, mTOR was further recruited on the lysosomes suggestive of further inhibition of autophagy, as shown through LC3-LAMP1 co-staining, and this synergized with the OCC CM-induced CAFs phenoconversion (Figure 5A, panels a–c). Conversely, rapamycin promoted the detachment of mTOR from the lysosomes, which resulted in the induction of autophagy and the concurrent inhibition of the CAFs phenoconversion (Figure 5A, panels a–c). Interestingly, when similar treatments were performed in CAFs, the inhibition of autophagy by proline maintained, and actually re-enforced, the CAF-phenotype while the induction of autophagy by rapamycin partially reversed CAFs activation (Figure 5B, panels a–c).

**Figure 6:**
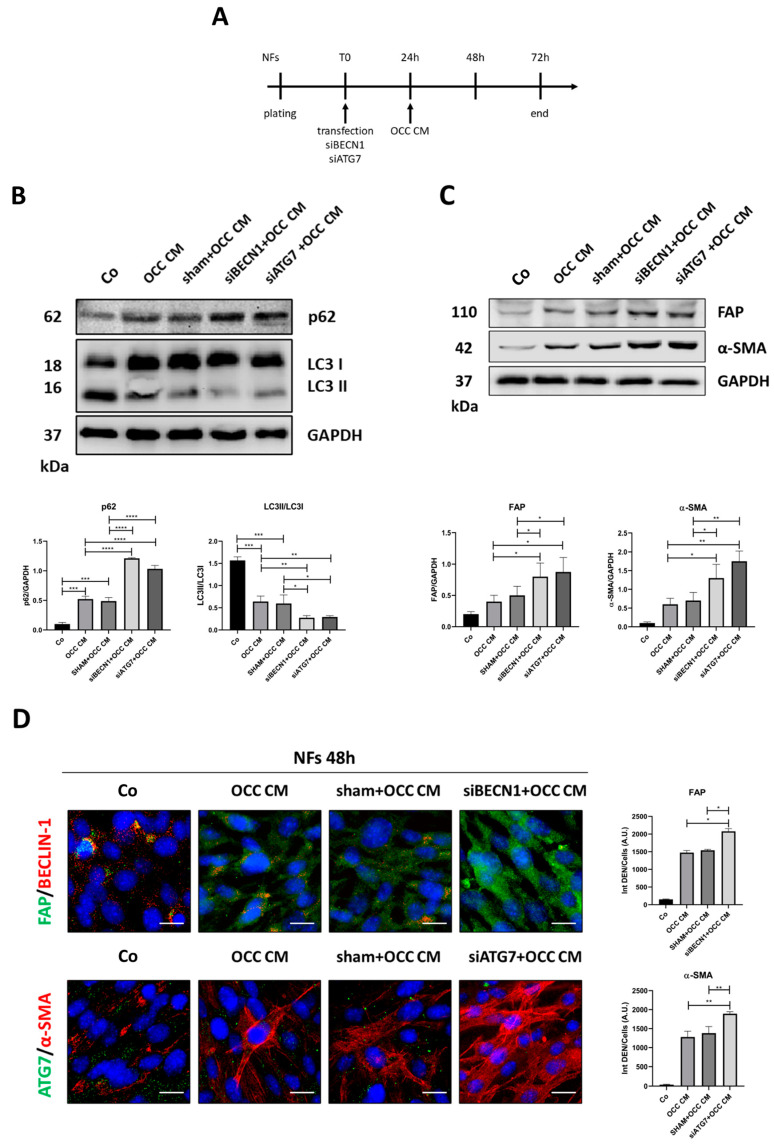
Autophagy-knockdown exacerbates CAF-phenotype. (**A**) Schematic representation of the experimental timeline, where each bar represents 24 h. NFs were silenced for *BECN1* or *ATG7* and, on the following day, were treated with OCC CM for further 48 h. (**B**) Cell homogenates were analyzed by means of Western blotting to monitor autophagy flux (p62 and LC3). The filter was probed with GAPDH as a loading control. The blot is representative of three replicates, and densitometric data are reported in the graphs. (**C**) The same homogenates were analyzed by means of Western blotting for assessing FAP and α-SMA expression. The filter was probed with GAPDH as a loading control. The blot is representative of three replicates, and densitometric data are reported in the graphs. (**D**) NFs were transfected and treated as reported in panel A before being fixed and stained. Immunofluorescence double staining for (i) FAP (green)—BECLIN-1 (red) and (ii) ATG7 (green)—α-SMA (red). Scale bar = 20 μm; magnification = 63×. All data are representative of different fields per condition. Average fluorescence intensity was calculated from at least 50 cells from randomly chosen fields. Statistical analysis was performed using GraphPad Prism 5.0 software. Bonferroni’s multiple comparison test after one-way ANOVA analysis (unpaired, two-tailed) was employed. Significance was considered as follows: **** *p* < 0.0001; *** *p* < 0.001; ** *p* < 0.01; * *p* < 0.05.

As further confirmation, we silenced *BECN1* and *ATG7*, two essential autophagy-related genes, in NFs prior to incubation with OCC CM for further 48 h (Figure 6A). Western blotting proved the successful silencing of BECLIN-1 and ATG7 (Supplementary Figure S2), and the concomitant inhibition of autophagy as indicated by the reduced LC3-II/LC3-I ratio (indicative of reduced autophagosome formation) and the accumulation of p62 (indicative of reduced cargo degradation) (Figure 6B). Importantly, we observed that autophagy knockdown exacerbated the CAF-phenotype, as shown by the increased expression of FAP and α-SMA (Figure 6C) and the stress fiber-like pattern (Figure 6D). Taken together, these data support the view that enhancing autophagy could counteract the activation of CAFs.

### 2.4. Resveratrol Reprograms CAFs to NFs

In previous work, we have shown that the nutraceutical resveratrol (RV) is a strong autophagy inducer [30], promotes several changes in the tumor microenvironment, and regulates the crosstalk between cancer cells and stroma [31,32,33]. Very recently, we have shown that RV hampers glucose uptake and glycolysis while restoring autophagy in ovarian cancer cells exposed to IL-6 [26].

**Figure 7:**
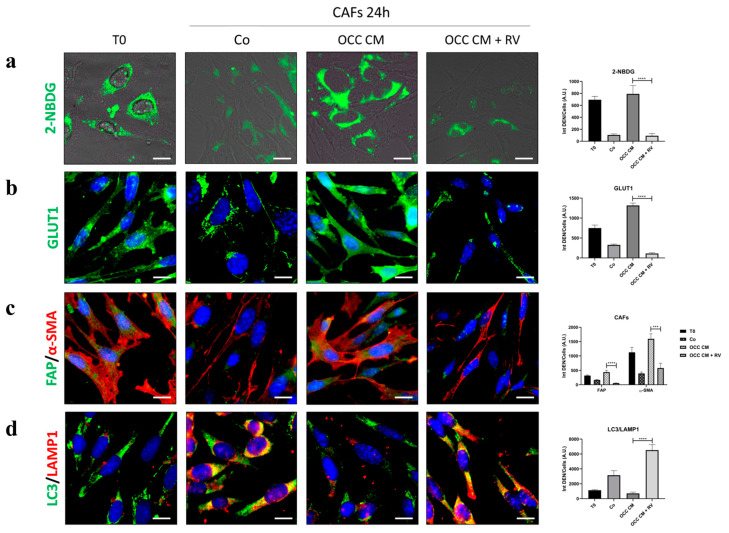
Resveratrol promotes the phenoreversion of the CAF-phenotype. CAFs were cultured with Co or OCC CM supplemented or not with 100 µM RV for 24 h. Cells were stained for 2-NBDG uptake (**a**), the expression of GLUT1 (**b**), FAP and α-SMA (**c**), and the formation of autolysosomes (LC3-LAMP1 co-localization) (**d**). The fluorescence quantifications are reported in the graphs. Scale bar = 20 μm; magnification = 63×. The images shown are representative of different fields for each condition. Average fluorescence intensity was calculated from at least 50 cells from randomly chosen fields. Statistical analysis was performed using GraphPad Prism 5.0 software. Bonferroni’s multiple comparison test after one-way ANOVA analysis (unpaired, two-tailed) was employed. Significance was considered as follows: **** *p* < 0.0001; *** *p* < 0.001.

Here, we tested the capability of RV to counteract the glucose consumption necessary for the maintenance of the CAFs’ activated phenotype. CAFs were cultured with OCC CM supplemented or not with RV for 24 h, and the changes were compared with those induced by control medium (Figure 7). In the presence of RV, the conditioning medium fails to promote the glucose uptake (panel a), in association with a loss of GLUT1 staining on the plasma membrane (panel b), and this is reflected in a strong reduction in FAP and α-SMA positivity (panel c) along with the restoration of the autophagy flux (panel d). These findings collectively indicate that RV can promote the phenoreversion of CAFs towards an NF-phenotype through the upregulation of autophagy.

### 2.5. Autophagy Upregulation Reprograms Myofibroblasts to NFs

**Figure 8:**
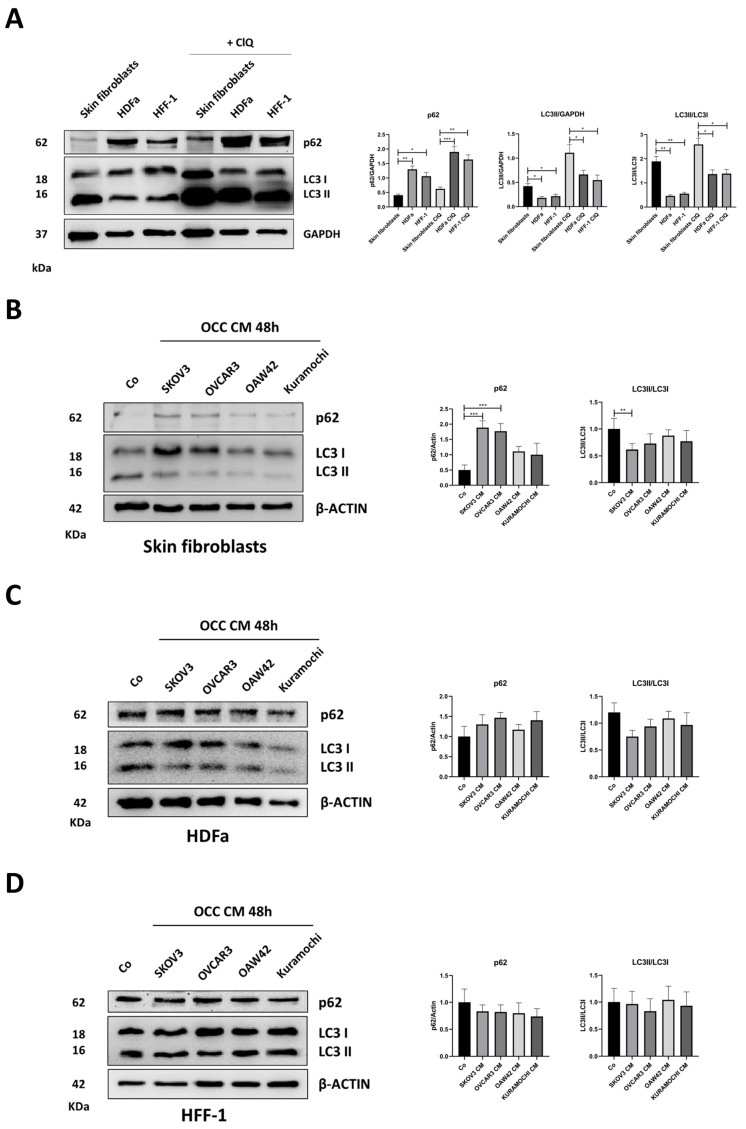
Skin fibroblasts, HDFa, and HFF-1 differ in their basal autophagy. (**A**) Skin fibroblasts, HDFa, and HFF-1 were cultured in the absence/presence of 30 µM chloroquine (ClQ) for 8 h. Cell homogenates were analyzed by means of Western blotting for the expression of the autophagic markers p62 and LC3. The filter was probed with GAPDH as a loading control. (**B**–**D**) The three fibroblast cell models were cultured with control medium (Co) or OCC CM collected from four different cancer cell lines (SKOV3, OVCAR3, OAW42, and Kuramochi) for 48 h, as previously mentioned. Cell homogenates from skin fibroblasts (**B**), HDFa (**C**), and HFF-1 (**D**) were analyzed by means of Western blotting for the expression of p62 and LC3. The filters were probed with β-actin as a loading control. Autophagy flux was monitored by the conversion rate of the cytosolic immature form LC3-I to the lipidated mature form LC3-II and by p62 levels. The densitometric data of the triplicates are reported in the graphs. Statistical analysis was performed using GraphPad Prism 5.0 software. Bonferroni’s multiple comparison test after one-way ANOVA analysis (unpaired, two-tailed) was employed. Significance was considered as follows: *** *p* < 0.001; ** *p* < 0.01; * *p* < 0.05.

Previous data demonstrate that RV could revert CAFs phenoconversion by concomitantly preventing the glycolytic shift and rescuing autophagy. Thus, we aimed to identify the possible mechanistic explanations for the lack of OCC CM-induced phenotypic changes in HDFa and HFF-1 cells. First, we monitored autophagy in the three fibroblast cell lines (Figure 8A). In basal conditions, we noticed that skin fibroblasts exhibit a higher autophagy flux compared to HDFa and HFF-1, as indicated by the low p62 expression along with the higher LC3-II/LC3-I ratio (which reflects the rate of conversion of LC3-I into LC3-II). Since the net accumulation of LC3-II in the cell is the result of the rate of synthesis and degradation, we inhibited the turnover of autophagosome by co-incubating the cells with chloroquine (ClQ). In this condition, the level of LC3-II reflects the amount of protein synthesized during the treatment. From the Western blotting shown in Figure 8A(right part), it is clearly apparent that LC3-II levels were higher in skin fibroblasts than those in HDFa and HFF-1 cells. Next, we assessed the modulation of autophagy flux in the three fibroblast cell models cultured with cancer cell secretions, as previously reported for Figure 1 and Figure 2. As shown in Figure 8B, the incubation with OCC CM (especially SKOV3 CM) inhibited autophagy in skin fibroblasts, as shown by the p62 accumulation and the decreased LC3-II/LC3-I ratio. On the other hand, the addition of OCC CM to HDFa and HFF1 cells (which are characterized by a myofibroblast-like phenotype and lower basal autophagy) did not elicit any significant change in p62 and the LC3-II/LC3-I ratio (Figure 8C,D). 

**Figure 9:**
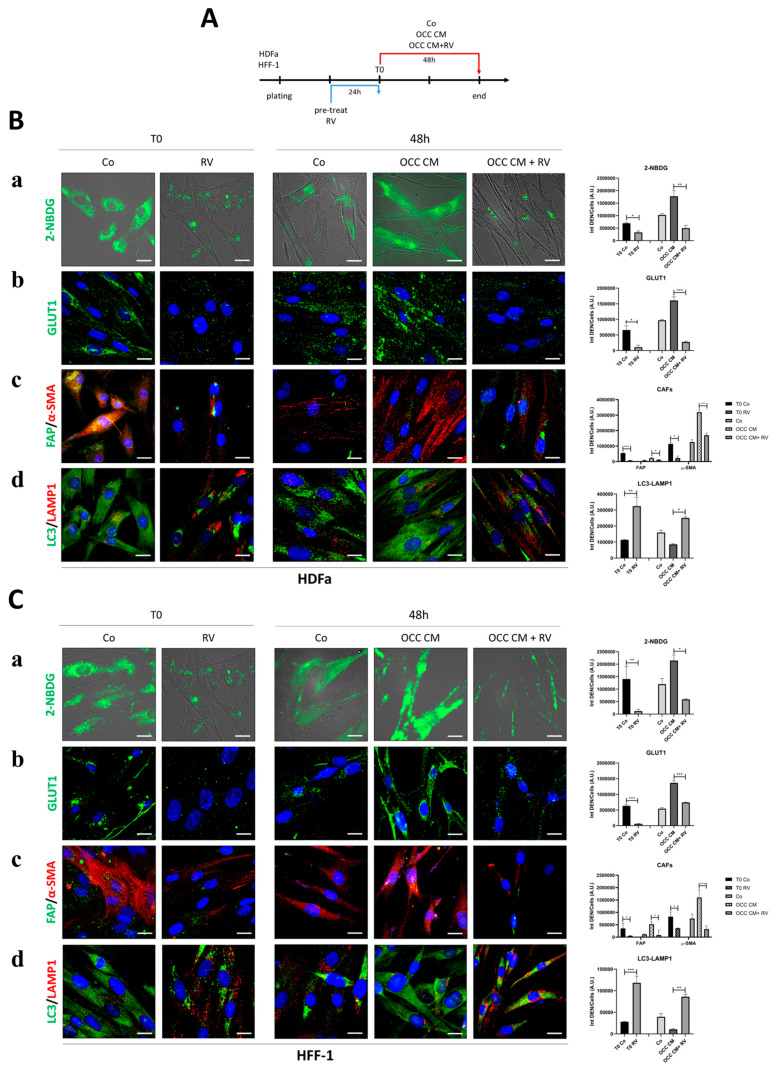
Resveratrol reprograms myofibroblasts to NFs and prevents their activation to CAFs. (**A**) Schematic representation of the experimental timeline. HDFa and HFF-1 cells were pre-treated with 100 µM RV for 24 h (T0) and then cultured with control medium (Co) or OCC CM supplemented or not with 100 µM RV for further 48 h. (**B**,**C**) Cells were stained for 2-NBDG uptake (**a**), the expression of GLUT1 (**b**), FAP and α-SMA (**c**), and the formation of autolysosomes (LC3-LAMP1 co-localization) (**d**). The fluorescence quantifications are reported in the graphs. Scale bar = 20 μm; magnification = 63×. The images shown are representative of different fields for each condition. Average fluorescence intensity was calculated from at least 50 cells from randomly chosen fields. Statistical analysis was performed using GraphPad Prism 5.0 software. Bonferroni’s multiple comparison test after one-way ANOVA analysis (unpaired, two-tailed) was employed. Significance was considered as follows: *** *p* < 0.001; ** *p* < 0.01; * *p* < 0.05.

Given that HDFa and HFF-1 were shown to be insensitive to OCC CM because of the high basal expression of activated fibroblast markers (e.g., FAP and α-SMA), we tested whether RV could revert their CAF-like phenotype into an NF-like phenotype through the upregulation of autophagy. We pre-treated the cells with RV for 24 h (T0) before cultivating them in OCC CM containing or not RV for further 48 h and analyzed the changes induced with respect to control medium (Figure 9A). We monitored the internalization of glucose by 2-NBDG uptake and GLUT1 localization, the expression of FAP and α-SMA, and the autophagosome-lysosome fusion using LC3-LAMP1 co-staining. 

The pre-treatment with RV strongly reduced the glucose uptake and the plasma membrane localization of GLUT1 in both HDFa and HFF-1 fibroblast cell lines (Figure 9B,C, panels a,b), and this was coupled with a significant decrease in FAP and α-SMA expression (panel c), suggestive of the reprogramming of myofibroblasts to NFs, in parallel with the induction of autophagy, as shown by the increased LC3-LAMP1 co-localization (panel d).

Finally, as a counterproof, the cells which had reverted to NF-like due to RV pre-treatment were then exposed for 48 h to Co, OCC CM, or OCC CM + RV. Strikingly, the cells maintained an NF-like phenotype, along with low glucose uptake and sustained autophagy, when cultured in control medium for 48 h, indicating that the beneficial effect of the RV pre-treatment could last for so long. However, upon incubation with OCC CM, the cells increased the uptake of glucose and acquired again the myofibroblast features (as shown by their positivity to FAP and α-SMA) along with the downregulation of autophagy (as indicated by diffused cytoplasmic LC3 staining). Once again, this latter effect was inhibited by RV. 

## 3. Discussion

Currently, one aspect of ovarian tumorigenesis which is still poorly addressed is how the tumor stroma becomes desmoplastic, a condition that negatively impacts prognosis [2,34,35]. 

Growth factors, cytokines, chemokines, metabolites, and other regulatory molecules in the CAFs’ secretome influence the evolution and biological function of the tumor microenvironment, thus impinging on ovarian cancer growth and progression and, eventually, on the clinical outcome. In more detail, CAFs play key roles in ovarian cancer malignancy through ECM remodeling, maintaining the stemness of cancer stem cells, and modulating tumor immunity and cancer cell metabolism [10,18,36].

Targeting CAFs poses a major challenge that greatly limits the efficacy of the therapeutic approaches, given their distinct context-dependent roles [37,38]. An alternative promising strategy to directly targeting a specific CAF-marker may be to disrupt a series of CAFs-rewiring mechanisms. Therefore, understanding the mechanisms underlining CAFs activation and exploiting them therapeutically is fundamental to uncover the key vulnerabilities of the tumor [39]. Besides cancer cells, stromal cells, and fibroblasts in particular, are also subjected to altered metabolism depending on the microenvironmental context, further increasing the heterogeneity and complexity of the cellular composition and cell behavior in the tumor microenvironment [21,37,38,39,40]. With their secretions, cancer cells corrupt the fibroblasts, which become activated fibroblasts (or CAFs), and these in turn undergo a metabolic switch to glycolysis, producing energy-rich metabolites that fuel the tumor mass [15,41].

Here, we report a novel pathway showing that the phenoconversion of normal (skin) fibroblasts into CAFs induced by the secretome of ovarian cancer cells involves the glucose-dependent inhibition of autophagy. Notably, we demonstrate that the inhibition of glycolysis by 2DG not only prevents CAFs activation, but, more importantly, results in the phenoreversion of CAFs to the NF-phenotype. HDFa and HFF-1 cells, basally showing the phenotype of activated fibroblasts, were not sensitive to the OCC secretome. HDFa cells were isolated from an adult subject and possibly underwent an increase in FAP and α-SMA in response to aging-related inflammation [42], while HFF-1 cells originated from the foreskin of a neonatal subject and are known to express higher myofibroblast-related markers compared to skin fibroblasts [43]. However, much like the CAFs derived from skin fibroblasts exposed to OCC CM, HDFa and HFF-1 were shown to revert their phenotype back to that of normal fibroblasts upon glucose restriction and autophagy induction. Our data reinforces the concept that glycolysis represents a priming event required for the acquisition and maintenance of the CAF-phenotype [13,23,24].

Our findings are in line with the view that the acquisition of the CAF-phenotype may not be permanent, but it is reversible. In our experience, the withdrawal of cancer cell-conditioning medium resulted in the spontaneous reversion of CAFs towards the NF-phenotype within 48–96 h. This phenomenon can be epigenetically regulated by microRNAs. It has been shown that ovarian cancer cells reprogram NFs to CAFs through the action of miRNAs (e.g., miR-31, miR-155, and miR-214), and this opens up promising therapeutic strategies [44,45]. Such phenotypic changes are associated with cell organelle and morphology remodeling, which requires the modulation of autophagy, a process of macromolecular degradation and the recycling of substrates for replacing cellular structures [21]. 

Several soluble factors abundantly present in the tumor microenvironment can regulate autophagy [17,18,21,46,47,48,49]. The de-regulation of autophagy has been recognized as one of the main metabolic features of both cancer cells and stroma subjected to environmental changes [17]. The mechanistic involvement of autophagy in CAFs reprogramming remains controversial, given its dichotomous role [21,50,51]. Autophagy in CAFs can either counteract cancer progression in the early stages of tumorigenesis or exert a tumor-promoting role in advanced stages [40]. Moreover, CAFs localized in different parts of the tumor mass may display different levels of autophagy depending on the microenvironmental conditions they experience (e.g., hypoxia, nutrient deprivation, acidic pH, DNA damage, mitochondrial, and ER stresses) [21,40]. This makes it difficult to attribute a unique and well-defined functional role to autophagy in CAFs activation and its contribution in tumor progression. 

Here, we show that the ovarian cancer cell secretome induces the downregulation of basal autophagy in normal skin fibroblasts, and this metabolic change is required for the acquisition and maintenance of the CAF-phenotype. In fact, knocking down the autophagy-related genes *BECN1* or *ATG7* exacerbates the upregulation of CAF-related markers promoted by the conditioning medium. We hypothesize that the main soluble factors present in the conditioning medium negatively impinging on autophagy may be IL-6 and LPA [26,46,47,48]. Overall, our observations are consistent with recent evidence showing that the autophagy inhibitor SAR405 induces the activation of NFs to CAFs [52]. 

Cancer cells rely on several integrated metabolic pathways that support their survival in response to changes in the microenvironment. Given that the metabolic pathways of glycolysis and autophagy are interlinked [53], we show that culturing normal skin fibroblasts with cancer cell-conditioning medium supplemented with proline (which inhibits autophagy) enhances and supports the maintenance of the CAF-phenotype while rapamycin (which induces autophagy) hinders CAFs phenoconversion. Of note, similar treatments on CAFs showed that proline reinforces CAF activation and, contrarily, rapamycin-induced autophagy promotes phenoreversion to the NF-phenotype.

Our group reported that the nutraceutical resveratrol (RV) elicits pleiotropic anticancer benefits in several cancer models by hampering glucose metabolism, reducing the secretion of pro-inflammatory cytokines, and concomitantly upregulating autophagy via different pathways [26,30,31,32,33,46,47,48]. Here, we demonstrate that RV counteracts the reprogramming associated with CAFs activation and promotes the phenoreversion of CAFs to NFs. Thus, using RV as well as a combination of therapeutic compounds acting on glycolysis and autophagy may represent a valuable strategy for targeting the metabolic rewiring associated with CAFs activation, which may synergize and enhance the efficacy of anti-cancer therapy. 

Cancer cell-conditioning medium failed to induce upregulation in myofibroblast cell models. Notably, RV was able to convert the phenotype of HDFa and HFF-1 cells (which basally display a low level of autophagy and an upregulated expression of FAP and α-SMA) into an NF-like phenotype and, more importantly, prevented their activation into CAFs when exposed to ovarian cancer cell-conditioned medium.

**Figure 10:**
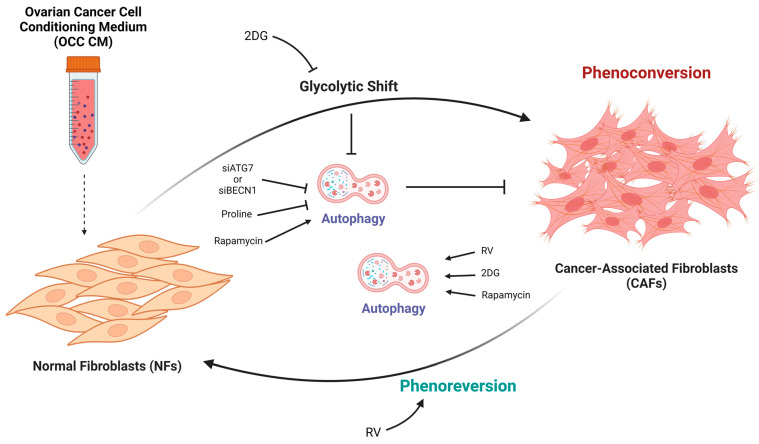
Schematic representation of the mechanisms involved in the metabolic rewiring of CAFs. The ovarian cancer cell-conditioning medium induces the glycolytic shift required for the phenoconversion and maintenance of the CAF-phenotype. Targeting the glycolytic reprogramming hampers CAFs activation, which relies on the glucose-dependent inhibition of autophagy. On the contrary, autophagy induction promotes the phenoreversion of CAFs to NFs.

Taken together, our data underline the fact that targeting the glucose-dependent inhibition of autophagy at different levels, either by inhibiting glycolysis or upregulating autophagy, represents a novel promising strategy to counteract CAFs activation and reprogram CAFs into resident normal fibroblasts (Figure 10). As mentioned above, reverting such a phenotype may dampen fibrosis and inflammation, and enhance cytotoxic immune cell infiltration and the concomitant improvement of drug delivery and therapy efficiency [37,39,54]. Additionally, given the prominent role of CAFs in orchestrating the intricate metabolic network within the tumor microenvironment, the present findings may have several implications in the crosstalk not only with cancer cells but also with other stromal components (e.g., immune cells) as well as influencing tumor growth, metastasis, and the response to cancer immunotherapy [55]. We are aware of the limitations of the present study, that should be solved in subsequent work. For instance, it is important to reproduce these observations in patient-derived samples (both cancer cells and CAFs). Also, we need to identify the soluble factors present in the OCC secretome responsible for the above effects. 

## 4. Materials and Methods

### 4.1. Cell Culture

All cell lines were maintained under standard culture conditions (37 °C, 5% CO_2_). Ovarian cancer cells (SKOV3 (cod. HTB-77; ATCC, Manassas, VA, USA), OVCAR3 (cod. HTB-161; ATCC), OAW42 (cod. 85073102; EACC, Porton Down, Salisbury, UK), and Kuramochi (cod. cod. JCRB0098; Japanese Collection of Research Bioresources Cell Bank; Ibaraki, Japan)), human skin fibroblasts (kindly provided by Prof. E. Grossini’s lab, Università del Piemonte Orientale, Novara, Italy; [56]), HDFa human adult dermal fibroblasts (cod. PCS-201-012; ATCC), and HFF-1 human foreskin fibroblasts (cod. SCRC-1041; ATCC) were cultured in RPMI 1640 medium (cod. R8758; Sigma Aldrich, St. Louis, MO, USA) supplemented with 10% heat-inactivated fetal bovine serum (FBS, cod. ECS0180L; Euroclone, Milan, Italy), 1% glutamine (cod. G7513; Sigma-Aldrich), and 1% penicillin/streptomycin solution (PES, cod. P0781; Sigma Aldrich) Table 1.

### 4.2. Reagents

The compound 2-deoxy-D-glucose (2DG, cod. D6134; Sigma Aldrich) is an inert non-metabolizable glucose analog. 2DG was dissolved in sterile water and used at a final concentration of 10 mM.

Proline (Pro, cod. P0380; Sigma Aldrich) supplementation was used as a negative feedback signal for autophagy activation (as shown in [28]). Pro was dissolved in sterile water and used at a final concentration of 2 mM.

Rapamycin (Rap, cod. R8781; Sigma Aldrich) is an inhibitor of mTOR and an autophagy inducer. Rap was dissolved in DMSO and used at a final concentration of 10 µM.

Resveratrol (RV, cod. R5010; Sigma Aldrich) is a nutraceutical compound that inhibits glucose uptake and induces autophagy [26]. RV was dissolved in DMSO and used at a final concentration of 100 µM.

Chloroquine (ClQ, cod. C6628; Sigma Aldrich) blocks autophagosome–lysosome fusion, thus impairing the autophagy flux. ClQ was dissolved in sterile water and used at a final concentration of 30 µM.

### 4.3. Antibodies

The following primary antibodies were employed for either immunofluorescence or Western blotting: mouse anti-α-SMA (1:500, cod. A5228; Sigma Aldrich), rabbit anti-FAP (1:50 for IF, 1:500 for WB, cod. MA5-32670; Invitrogen, Waltham, MA, USA), mouse anti-β-actin (1:2000, cod. A5441; Sigma Aldrich), rabbit anti-GAPDH (1:1000, cod. G9545; Sigma Aldrich), mouse anti-HK2 (1:100 for IF, 1:500 for WB, cod. NBP2-02272; Novus Biologicals, St. Charles, MI, USA), rabbit anti-mTOR (1:100 for IF, 1:1000 for WB, cod. 2983; Cell Signaling Technology, Danvers, MA, USA), mouse anti-LAMP1 (1:1000, cod. 555798; BD Biosciences, Franklin Lakes, NJ, USA), rabbit anti-LC3 (1:1000, cod. L7543; Sigma Aldrich), mouse anti-LC3 (1:100, cod. 0231-100/LC3-5F10; Nanotools, Teningen, Germany), rabbit anti-ATG7 (1:500, cod. AB10511; Millipore, Burlington, MA, USA), mouse anti-BECLIN1 (1:250, cod. 612112; BD Biosciences), rabbit anti-p62 (1:500, cod. 8025; Cell Signaling Technology), mouse anti-p62 (1:1000, cod. MABC32; Millipore), and rabbit anti-GLUT1 (1:50, cod. 07-1401; Millipore).

### 4.4. Collection of the Ovarian Cancer Cell-Conditioning Medium (OCC CM)

Ovarian cancer cells (namely, SKOV3, OVCAR3, OAW42, and Kuramochi) were grown in 75 cm^2^ flasks to reach 90–95% confluency in RPMI-1640 (containing 10% FBS), and the conditioning medium (OCC CM) was collected at 48 h. OCC CM was centrifuged at 2000 rpm for 5 min to remove cell debris, diluted 70:30 (OCC CM: RPMI-1640), and then stored at −20 °C until use.

### 4.5. Glucose Uptake Assay

2-NBDG (cod. N13195; Life Technologies, Paisley, UK), a well-known fluorescent glucose analog that has been employed to monitor glucose uptake in living cells, was dissolved in sterile water and used at a final concentration of 50 µM. Cells were seeded onto coverslips (5000 cells/cm^2^), allowed to adhere, and treated as indicated. The staining was performed as previously described in [26]. Coverslips were acquired immediately using a fluorescence microscope (DMI6000; Leica Microsystems, Wetzlar, Germany). 

### 4.6. Cell Transfection 

Cells were plated on coverslips or on Petri dishes depending on the experiment performed (5000 cells/cm^2^ for IF; 10,000 cells/cm^2^ for WB). The siRNA-loaded liposomal complexes were prepared in Opti-MEM I Reduced Serum Medium (cod. 11058021, Life Technologies) with 150 pmol of either siATG7 or siBECN1 and Lipofectamine 3000 (cod. L3000-015, Life Technologies, Carlsbad, CA, USA). After 6 h of incubation, the medium was replaced with complete culture medium; on the following day, treatments were performed as indicated. The negative control of the transfection experiments is represented by the siRNA scramble control (referred to as sham). The sequences of the siRNA used are the followings: siRNA scramble: 5′-AGGUAGUGUAAUCGCCUUGTT-3′; siRNA ATG7: 5′-GGGUUAUUACUACAAUGGUGTT-3′; siRNA BECN1: 5′-GGAACUCACAGCUCCAUUACUUACCAC-3′. 

### 4.7. Western Blotting 

Cells were plated on Petri dishes (10,000 cells/cm^2^) and treated or transfected as indicated. Standard procedure was used for preparing cell homogenates, and Western blotting was performed as detailed in [47]. The bands were detected with Enhanced Chemiluminescence reagents (ECL, cod. NEL105001EA; Perkin Elmer, Waltham, MA, USA) and imaged with the VersaDOC imaging system. For loading control, the filters were re-probed with β-actin or GAPDH. Densitometric analysis was performed with Quantity One software (v.4.5).

### 4.8. Immunofluorescence 

Cells were plated on sterile coverslips (5000 cells/cm^2^) and treated or transfected as indicated. Standard procedure was used for immunofluorescence, as detailed in [47]. The coverslips were incubated overnight at 4 °C with specific primary antibodies and, on the following day, were incubated with dye-conjugated secondary antibodies for 1 h at room temperature. Nuclei were stained with the UV fluorescent dye DAPI (4′,6-diamidino-2-phenylindole). Coverslips were mounted onto glasses using SlowFade reagent (cod. S36936; Invitrogen) and imaged under a fluorescence microscope (Leica DMI6000). Integrated fluorescence values (Int DEN) were determined using the ImageJ software (v. 1.48; NIH). For co-localization assays, the graphs show the quantification of yellow fluorescence density obtained by the merging of green and red channels (representing the close proximity between mTOR/HK2, mTOR/LAMP1, and LC3/LAMP1).

### 4.9. Imaging Acquisition and Analysis

Fluorescence images were acquired using a fluorescence microscope (Leica DMI6000). For each experimental condition, at least three slides were prepared in separate experiments, and five to ten randomly chosen microscopic fields were imaged by two independent investigators unaware of the treatment. Representative images of selected fields are shown.

### 4.10. Co-Immunoprecipitation

Cells were plated on Petri dishes (25,000 cells/cm^2^) and treated as indicated. Before collection, cells were incubated for 15 min with 1 mM of the chemical cross-linker 3-3′-dithiodipropionic acid di-(N-hydroxysuccinimide ester) (DTSP, cod. D3669, Sigma Aldrich). Cells were collected as detailed above and the protein content was measured using a BCA assay. The same amount of protein (500 μg) was incubated with the anti-mTOR antibody (5 μg), and to capture the immunocomplexes, 50 μL of Sepharose G beads (cod. 17061801; Cytiva, Uppsala, Sweden) was added to each sample. The immunocomplexes were then precipitated by means of centrifugation and eluted with Leammli buffer. Samples were loaded on an SDS-PAGE and immunoblotted with specific antibodies to assess the interaction mTOR-HK2.

### 4.11. Statistical Analysis

All data refer to at least three separate experiments. Data in histograms are shown as average ± S.D. Statistical analysis was performed with GraphPad Prism 5.0 software. Bonferroni’s multiple comparison test after one-way ANOVA analysis (unpaired, two-tailed) was employed. T-test analysis (unpaired, two-tailed) was employed in the experiments that required the comparison of two groups (control vs. treatment). Significance was considered as follows: **** *p*< 0.0001; *** *p* < 0.001; ** *p* < 0.01; * *p* < 0.05; not significant (ns) *p* > 0.05.

## Figures and Tables

**Table 1:** Genetic background of the ovarian cancer cell lines employed for obtaining the conditioning medium.

Cell Line	*BECN1*	*TP53*	*PTEN*	*AKT*	*PI3KCA*
SKOV3	Wild-type	Null	Wild-type	Wild-type	Mutated
OVCAR3	Wild-type	Mutated	Null	Wild-type	Wild-type
OAW42	Wild-type	Wild-type	Wild-type	Wild-type	Mutated
Kuramochi	Wild-type	Mutated	Wild-type	Mutated	Wild-type

## Data Availability

Data is contained within the article and Supplementary Materials.

## Supplementary Materials

The following supporting information can be downloaded at: https://www.mdpi.com/article/10.3390/ijms25115691/s1.
