# Supplementary material for: Ovarian Cancer Cell-Conditioning Medium Induces Cancer-Associated Fibroblast Phenoconversion through Glucose-Dependent Inhibition of Autophagy
Source: Int J Mol Sci. 2024 May 23;25(11):5691. doi: 10.3390/ijms25115691 (PMC11171902; doi:10.3390/ijms25115691)
Supplement: Supplementary file 1 [file ijms-25-05691-s001.zip › ijms-2948279-supplementary.pdf]

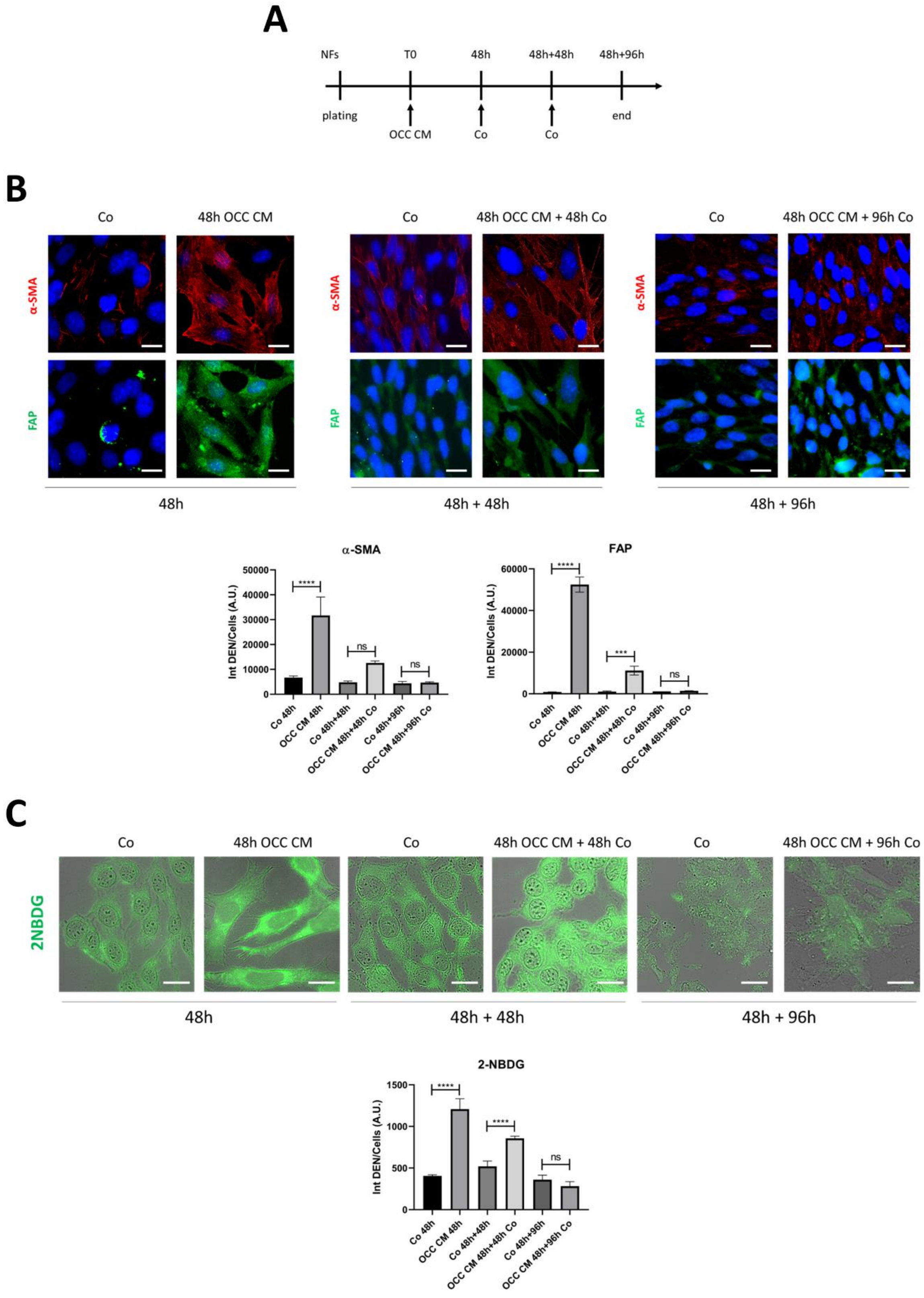

Supplementary Figure S1. CAF-phenotype requires the presence of cancer cell-conditioning medium. NFs were cultured with OCC CM for 48h, then OCC CM was replaced by control medium (Co) for the following 48h and 96h. A) Cells were fixed and stained for α-SMA (red) and FAP (green). B) Living cells were stained with 2-NBDG to assess glucose uptake. The images were acquired with the fluorescence microscope. Scale bar = 20 μm; magnification = 63×. Statistical analysis was performed with GraphPad Prism 5.0 software. Bonferroni's multiple comparison test after one-way ANOVA analysis (unpaired, two-tailed) was employed. Significance was considered as follow: **** $p < 0.0001$; *** $p < 0.001$; not significant (ns) $p > 0.05$.

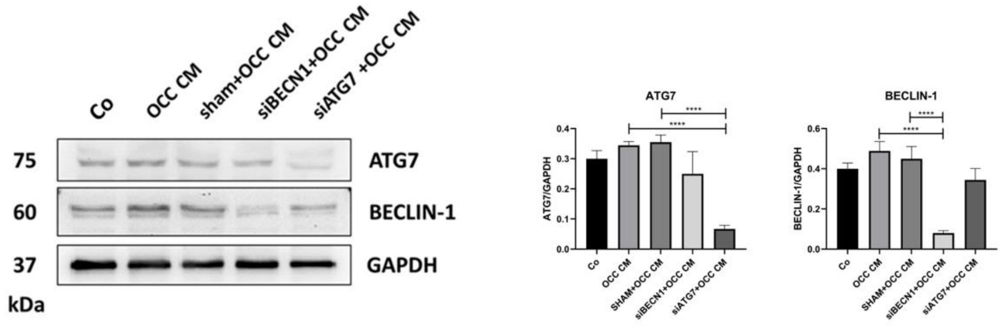

Supplementary Figure S2. Assessment of autophagy gene silencing. NFs were silenced for *BECN1* or *ATG7* and then the following day were treated with OCC CM for further 48h. Cell homogenates were analyzed by Western blotting for the expression of BECLIN-1 and ATG7 to monitor the efficiency of gene silencing. The filter was probed with GAPDH as a loading control. Densitometric data are reported in the graphs. Statistical analysis was performed by using GraphPad Prism 5.0 software. Bonferroni's multiple comparison test after one-way ANOVA analysis (unpaired, two-tailed) was employed. Significance was considered as follows: **** $p < 0.0001$.
